# The Effect of Self-Esteem on Attitudes Toward Aging in the Elderly

**DOI:** 10.7759/cureus.76899

**Published:** 2025-01-04

**Authors:** Elif Özcan Tozoğlu, Nilifer Gürbüzer

**Affiliations:** 1 Department of Psychiatry, University of Health Sciences, Erzurum Faculty of Medicine, Erzurum, TUR

**Keywords:** aging attitude, elderly persons, physical change, psychosocial growth, psychosocial loss, self-esteem etc

## Abstract

Objective

People's attitudes about aging are formed in early childhood, develop in adulthood, and are fully shaped in old age. Our study aimed to investigate the effect of self-esteem on attitudes toward aging in the elderly.

Method

A total of 264 people over the age of 65 without any known psychiatric or neurologic disease were included in the study. The sociodemographic data form, Rosenberg Self-Esteem Scale (RSES) with 12 sub-dimensions, and Attitude to Aging Questionnaire (AAQ) with three sub-dimensions were applied to each participant. Pearson correlation analysis was used to evaluate the relationship between quantitative variables. Hierarchical multiple regression analysis was used to determine the factors predicting the attitude toward aging.

Results

A moderately significant negative relationship was found between the AAQ sub-dimension of psychosocial loss score and RSES sub-dimension self-esteem (r=-0.340, p=0<0.001), continuity of self-concept (r=-0.258, p<0.001), depressive affect (r=-0.423, p<0.001), and psychosomatic symptoms (r=-0.311, p<0.001). Self-esteem was found to be predictive of attitude toward aging. When sociodemographic factors were controlled, it was found that 25.6% of the variance in attitude toward psychosocial loss (R^2^ change=25.6; p<0.001), 30.5% of the variance in attitude toward physical change (R^2^ change=30.5; p<0.001), and 34.9% of the variance in attitude toward psychosocial development (R^2^ change=34.9; p<0.001) could be explained by the specified sub-dimensions of RSES.

Conclusion

In our study, it was observed that self-esteem shaped from childhood to the present day is at least as effective as sociodemographic characteristics in the attitude toward aging.

## Introduction

Old age is an inevitable stage of life that every living being experiences. International organizations define the period of old age as 65 years and older. However, old age varies in biological, psychological, physical, and sociological dimensions [1]. Problems such as loss of job, loss of social roles, and decrease in income level may occur with aging. In addition, the loss of friends and family and difficulties in activities of daily living are important changes for older individuals. Most elderly individuals adapt to these changes, but those who cannot adapt may experience psychiatric symptoms. In addition to the loss of intelligence, memory, and senses in old age, longing for the past and unrealized ideals are symptoms of psychological aging. This is closely related to feeling and accepting oneself as old. Who is called old also depends on the age and attitude of the individual. Age is not just a number; it is influenced by many factors, such as emotional and physical health, socioeconomic status, culture, and ethnicity.

In old age, people acquire more and different life experiences, and the accumulation of these experiences makes the older individual different from other age groups. Young individuals tend to accept their ideas about the attitude of aging without questioning them, and this perception turns into their own attitudes toward aging when they get older [2]. Although old age is usually associated with losses, it is a multidimensional life stage in which there are gains and losses. Ericson calls this stage the wisdom stage when the individual accepts their own life and accepts death [3]. Cohen defines old age as an important period associated with reaching psychological maturity by evaluating previous life experiences and the final development of the human brain [4].

Attitudes toward aging are related to behavioral states such as knowledge, activities, and choices and experiences such as hopes, fears, and emotions. It is related to many aspects, such as work status, health, mobility, leisure activities, family relationships, character, fears, social participation, and sexual life. People's attitudes about old age are formed in early childhood, develop in adulthood, and are fully shaped in old age [5]. This attitude may change in a negative direction with the change in psychosocial status, in which old age is seen as a negative experience involving psychological and social losses; it may change more negatively with the health problems and physical changes that come with age or it may change positively with the wisdom and developmental experiences gained with aging. In other words, the attitude toward aging is shaped by both one's own internal world and what one receives from the external world.

It is thought that self-esteem, which is shaped from childhood and adolescence, may also affect the attitude toward aging. Self-esteem is defined as the acceptance and adoption of one's own abilities and strengths as they are a result of self-recognition and realistic evaluation of oneself and expresses the feelings of love, respect, and trust that one feels toward oneself [6]. Self-esteem is a constantly changing and learned concept that covers a whole life process. This learning process involves the interaction of the individual with their social environment, mainly with their family. According to Branden, self-esteem refers to a person's self-confidence and sense of satisfaction about oneself [7]. In other words, it is how one thinks and feels about oneself. A healthy individual, in terms of self-esteem, feels valuable and trusts that they can cope with problems in life. They have a positive and realistic view of themselves and their abilities. When things go wrong, they can accept themselves with their mistakes and feel valuable.

People with low self-esteem doubt their abilities and have unrealistic expectations about themselves [7]. Their ideas about their own worth are heavily influenced by other people's opinions, and they criticize themselves mercilessly. They tend to find nothing satisfying enough. A person's self-esteem is influenced by their (inner) conversations with themselves. Unlike other living beings, humans are aware of their own existence and question it. In social life, a person's communication with other people and the feedback they receive as a result of this communication are effective in forming their ideas about themselves. In order to understand a person, it is necessary to have an idea about that person's evaluation of themselves. For a person, their evaluation of themselves is almost as important as a matter of life and death. In response to every question directed to the person or in every situation where they have to make a decision, the answer or decision they will give will include their view of themselves [7]. Rosenberg considered self-esteem a positive or negative attitude toward oneself [8]. If a person has a positive attitude in evaluating themselves, their self-esteem is considered to be high, and if they have a negative attitude, their self-esteem is considered to be low [8].

Declining birth rates and longer life expectancy are increasing the proportion of older people in society. There are 617 million elderly people worldwide, constituting 8.5% of the general population. While the elderly population in Turkey was 7,186,204 people in 2018, it increased by 21.4% in the last five years and reached 8,722,806 people in 2023. The proportion of the elderly population in the total population increased from 8.8% in 2018 to 10.2% in 2023. Turkey ranks 67th out of 184 countries in terms of the proportion of elderly population [9]. If the proportion of the elderly population in the society is between 7% and 10%, the society is called an elderly society, and Turkey falls into this category. It is predicted that the proportion of elderly individuals in the total population will be 12.9% in 2030, 16.3% in 2040, 22.6% in 2060, and 25.6% in 2080 [9]. For this reason, studies on old age are becoming increasingly important.

However, studies in this field are less than expected. The basis of psychologically healthy aging is perhaps determined by the personality traits and self-esteem established during childhood and youth. The effect of old age spent with healthy and positive attitudes on both the elderly population, which is gaining a larger place in the population day by day, and the mental health of those living with them cannot be denied. For this reason, to make this attitude healthier and more positive, it is first necessary to determine what influences this attitude. In our study, we aimed to investigate whether the attitude of aging would be related to issues such as self-concept, trust in people, sensitivity to criticism, parental interest, and relations with the father, which are effective in determining self-esteem.

## Materials and methods

Sample and participants

The study will use criterion sampling, one of the purposive sampling methods. This type of sampling is based on a sample that meets certain predetermined criteria. The criteria determined by the researcher to explain the situations examined can be used for this type of sampling. In criterion sampling, the individuals planned to be included in the study are determined according to certain criteria. The criteria may be created by the researcher, or a previously prepared list of criteria can be used [10].

The study was planned to include people who applied to the psychiatry outpatient clinic between March 2024 and May 2024, who applied to obtain a report of mental competence for purposes such as obtaining a gun license, making transactions at the notary, who were over 65 years of age, who had no known psychiatric or neurological disease, who did not have a physical condition that would require a disability report, who had a Standardized Mini-Mental State Examination (SMMSE) score above 23 points, who had no active psychopathology in the examination performed by the psychiatrist by applying the Structured Clinical Interview for DSM-5 disorders (SCID-5), and who agreed to participate in the study. The following sociodemographic data form and scales will be applied to the people who agree to participate in the study.

Ethics and consent

The research protocol was approved by the Scientific Research Ethics Committee of Health Sciences University Erzurum Faculty of Medicine (Erzurum, Turkey) with the decision numbered BAEK 2024/03-55 and carried out in accordance with the Helsinki Declaration. Written informed consent was obtained from all participants, stating that they agreed to participate in the study and gave their consent to the publication of all clinical and other data contained in the manuscript.

Data collection tools

Sociodemographic Form for the Elderly

The data collection form for the elderly was created by the researcher. Studies were examined, factors that would affect the perception of old age of the elderly were determined, and items such as age, gender, marital status, having children, living with an elderly person before, current health status, and comorbidities were included in the form.

Standardized Mini-Mental State Examination

The SMMSE, which assesses cognitive status, was developed by Folstein et al. in 1975 [11]. Its validity and reliability in the diagnosis of mild dementia for the Turkish population was performed by Güngen et al. [12]. The SMMSE is categorized under five main headings: orientation (10 points), recording memory (3 points), attention and calculation (5 points), recall (3 points), and language (9 points). The scale is scored out of a total of 30 points and has two different variants for educated and uneducated people. Traditionally, scores between 24 and 30 are considered normal.

Structured Clinical Interview for DSM-5 Disorders (SCID-5/CV)

The SCID-5 is one of the most widely used diagnostic tools in clinical research worldwide. The latest version is SCID-5/CV, which is a comprehensive, standardized tool for the evaluation of major psychiatric disorders according to the definitions and criteria of DSM-5. This structured clinical interview includes 32 diagnostic categories with detailed diagnostic criteria and 17 diagnostic categories with research questions. The validity and reliability study of the Turkish version of SCID-5/CV was conducted [13].

Rosenberg Self-Esteem Scale (RSES)

The RSES was developed by Morris Rosenberg in 1965 [8]. The Turkish validity and reliability study was conducted by Füsun Çuhadaroğlu in 1986, and the reliability coefficient was found to be 0.75 and alpha value 0.65 [14]. This scale consists of 63 items and has 12 subscales. These subscales separately assess self-esteem, continuity of self-concept, trust in people, sensitivity to criticism, depressive mood, dreaminess, psychosomatic symptoms, feeling of threat in interpersonal relationships, degree of ability to participate in discussions, parent-child relationship, relationship with the father, and psychic isolation. According to the evaluation system within the scale, the subjects score between 0 and 6. In comparisons made with numerical measurements, self-esteem is evaluated as high (0-1 point), medium (2-4 points), and low (5-6 points).

Attitude to Aging Questionnaire (AAQ)

The AAQ was created by Laidlaw et al. and the WHOQOL-OLD Group in 2007 [15], and a Turkish adaptation study was conducted by Eser et al. in 2011 [16]. The questionnaire includes psychosocial loss, physical change, and psychosocial growth sub-dimensions. To assess attitudes toward psychosocial loss, items include seeing aging as a time of loss, seeing oneself as alienated or excluded from society, etc. To assess physical change, some items measure how aging reduces energy and how sports and physical activities can no longer be performed as before. To assess psychosocial growth, some items include statements that aging is a period of mental maturation and a period in which they can transfer their experiences. Each question is evaluated with a five-point Likert-type response scale ranging from 1 to 5. After the psychosocial loss dimension score is inverted, as the scale score increases, the attitude score of the related dimension also changes positively. A minimum score of 8 and a maximum score of 40 can be obtained from each dimension.

Statistical methods

Analyses were conducted using IBM SPSS Statistics for Windows, Version 26 (Released 2019; IBM Corp., Armonk, New York). Data were presented in terms of mean, standard deviation, and count. A normality analysis was performed to check whether the skewness and kurtosis values of all variables fall within the range of -2 to +2. These values indicate that the normality assumption is met [17]. Data were presented as mean, standard deviation, percentage, and number. An independent sample t-test was used since a normal distribution condition was provided in comparisons between two independent groups. Pearson correlation analysis was used to evaluate the relationship between quantitative variables, and hierarchical multiple regression analysis was used to determine the factors predicting the dependent variable. The sub-dimensions of the AAQ were considered separately as the dependent variable. Qualitative data from independent variables were dummy-coded (0 = no, 1 = yes). Independent variables (qualitative and quantitative data) were individually linearly regression analyzed with the dependent variable. Hierarchical regression analysis was performed by creating a model with qualitative and quantitative data that were significant as a result of the analysis. A p-value of 0.05 was considered statistically significant in all statistical analyses.

## Results

Sociodemographic characteristics of the participants

A total of 264 participants (168 women and 96 men) were included in the study. The mean age of the participants was 70.56±6.28 years. Analysis revealed that 83% of the participants were married, 9.1% had a deceased spouse, 5.7% had no children, 18.2% were university graduates, and 52.3% were not working. Approximately 54.5% of the participants had at least one chronic disease, and 79.5% had a history of living with an elderly person in their youth. The sociodemographic characteristics and scale scores of the participants are shown in Table 1.

**Table 1:** Descriptive variables for the participants SD: standard deviation

Characteristics		n (%)	Mean±SD
Age	Both men and women	264 (100)	70.56±6.28
Gender	Women	168 (63.6)	-
	Men	96 (36.4)	-
Marital status	Married	219 (83)	-
	Single/divorced	21 (7.9)	-
	Widowed	24 (9.1)	-
Education status	Primary education	162 (61.4)	-
	High school	54 (20.4)	-
	University	48 (18.2)	-
Employment status	Not working	138 (52.3)	-
	Working	18 (6.8)	-
	Retired	108 (40.9)	-
Income	Under 17000 TL	136 (51.5)	-
	17000-30000 TL	92 (34.8)	-
	Over 30000 TL	36 (13.5)	-
Child status	There is	249 (94.3)	-
	No	15 (5.7)	-
Cigarette smoking	Yes	30 (11.4)	-
	No	234 (88.6)	-
Alcohol use	Yes	5 (1.9)	-
	No	259 (98.1)	-
Presence of chronic disease	Yes	144 (54.5)	-
	No	120 (45.5)	-
Region of residence	Urban	186 (70.5)	-
	Rural	78 (29.5)	-
Living with the elderly in youth	Yes	210 (79.5)	-
	No	54 (20.5)	-
Attitudes on Aging Questionnaire (AAQ)	Psychosocial loss	-	26.81±6.47
	Physical change	-	23.84±5.76
	Psychosocial growth	-	25.73±3.99
Rosenberg Self-Esteem Scale (RSES)	Self-esteem	-	0.95±0.62
	Continuity of self-concept	-	2.64±1.28
	Trust in people	-	1.27±0.62
	Sensitivity to criticism	-	2.14±0.92
	Depressive affect	-	1.57±1.27
	Dreaminess	-	0.41±0.75
	Psychosomatic symptoms	-	3.98±2.68
	Feeling threatened in interpersonal relationships	-	1.27±1.14
	Degree of participation in discussions	-	0.80±0.84
	Parental interest	-	2.00±1.69
	Relationship with the father	-	1.39±1.38
	Psychic isolation	-	0.50±0.62

AAQ and RSES scores according to gender and age

Gender differences were observed in AAQ and RSES scores. The psychosocial growth subscale of the AAQ was significantly higher in men than in women (p=0.015). When the sub-dimensions of the RSES were examined, self-esteem (p=0.020), continuity of self-concept (p<0.001), sensitivity to criticism (p=0.002), psychosomatic symptoms (p<0.001), feeling threatened in interpersonal relationships (p<0.001), and psychic isolation (p<0.001) sub-dimension scores were significantly higher in women compared to men. The relationship between scale scores and gender is shown in Table 2.

**Table 2:** Gender differences in Attitude to Aging Questionnaire and Rosenberg Self-Esteem Scale scores p<0.05: statistical significance level; SD: standard deviation; t: independent samples t-test

Parameters		Women	Men	t-value	p-value
		Mean±SD	Mean±SD		
Attitudes on Aging Questionnaire (AAQ)	Psychosocial loss	26.82±6.69	26.81±6.08	0.011	0.991
	Physical change	23.39±6.17	24.63±4.91	-1.677	0.095
	Psychosocial growth	25.25±3.64	26.56±4.45	-2.456	0.015
Rosenberg Self-Esteem Scale (RSES)	Self-esteem	1.02±0.65	0.83±0.55	2.332	0.020
	Continuity of self-concept	3.00±1.26	2.00±1.07	6.559	<0.001
	Trust in people	1.14±0.58	1.50±0.62	-4.625	<0.001
	Sensitivity to criticism	2.29±0.75	1.88±1.12	3.212	0.002
	Depressive affect	1.68±1.29	1.38±1.23	1.876	0.062
	Dreaminess	0.32±0.71	0.56±0.79	-2.466	0.015
	Psychosomatic symptoms	4.50±2.83	3.06±2.12	4.680	<0.001
	Feeling threatened in interpersonal relationships	1.50±1.18	0.88±0.93	4.740	<0.001
	Degree of participation in discussions	0.54±0.73	1.25±0.83	-6.992	<0.001
	Parental interest	1.75±1.58	2.44±1.81	-3.106	0.002
	Relationship with the father	1.14±1.33	1.81±1.39	-3.870	<0.001
	Psychic isolation	0.61±0.62	0.31±0.59	3.849	<0.001

A moderately significant negative correlation was found between the age of the participants and the AAQ subscale psychosocial loss score (r=-0.307, p=0<0.001) and physical change score (r=-0.277, p<0.001). The relationship between AAQ scores and the age of the participants is shown in Table 3.

**Table 3:** The relationship between participants' age and Rosenberg Self-Esteem Scale Scale scores and Attitude to Aging Questionnaire scores Note: p<0.05: statistical significance level; r: Pearson correlation coefficient

Parameters		Attitude to Aging Questionnaire		
		Psychosocial Loss	Physical Change	Psychosocial Growth
Age	r	-0.307	-0.277	-0.135
	p	0.000	0.000	0.029
Self-esteem	r	-0.340	-0.282	-0.093
	p	0.000	0.000	0.132
Continuity of self-concept	r	-0.258	-0.372	-0.046
	p	0.000	0.000	0.456
Trust in people	r	0.172	0.256	0.150
	p	0.005	0.000	0.015
Sensitivity to criticism	r	-0.206	-0.181	-0.262
	p	0.001	0.003	0.000
Depressive affect	r	-0.423	-0.607	-0.364
	p	0.000	0.000	0.000
Dreaminess	r	-0.295	-0.291	-0.100
	p	0.000	0.000	0.106
Psychosomatic symptoms	r	-0.311	-0.377	-0.307
	p	0.000	0.000	0.000
Feeling threatened in interpersonal relationships	r	-0.300	-0.317	-0.144
	p	0.000	0.000	0.014
Degree of participation in discussions	r	0.211	0.040	0.051
	p	0.001	0.515	0.409
Parental interest	r	0.085	0.180	0.414
	p	0.167	0.003	0.000
Relationship with the father	r	-0.195	0.284	0.188
	p	0.001	0.000	0.002
Psychic isolation	r	0.017	-0.213	-0.211
	p	0.784	0.000	0.001

Correlation results between AAQ and RSES scores

A moderately significant negative relationship was found between the AAQ sub-dimension of psychosocial loss score and RSES self-esteem (r=-0.340, p=0<0.001), continuity of self-concept (r=-0.258, p<0.001), depressive affect (r=-0.423, p<0.001), and psychosomatic symptoms (r=-0.311, p<0.001). The relationship between the participants' AAQ scores and RSES scores is shown in Table 3.

Analysis results on factors predicting attitude toward aging

Hierarchical multiple regression analysis was performed to determine the factors predicting participants' attitudes toward aging (for the three subscales of the AAQ). The sub-dimensions of AAQ were considered separately as dependent variables. Qualitative data from independent variables were dummy-coded (0 = no, 1 = yes). Independent variables (qualitative and quantitative data) were individually linearly regression analyzed with the dependent variable. Hierarchical regression analysis was performed by creating a model with qualitative and quantitative data that were significant as a result of the analysis. In the first step, the sociodemographic data determined for each of the three sub-dimensions of the AAQ and the RSES sub-dimensions determined in the second step were included in the model. The analysis results regarding the evaluation of the model are given in Table 4.

**Table 4:** Analysis results regarding the evaluation of the models Note: p<0.05: Statistical significance level a. Predictor: (Constant); age, marital status, education level, presence of chronic diseases, living with elderly when young, region of residence b. Predictor: (Constant); age, marital status, educational level, presence of chronic disease, living with the elderly when young, region of residence, self-esteem, continuity of self-concept, trust in people, sensitivity to criticism, depressive affect, dreaminess, psychosomatic symptoms, feeling threatened in interpersonal relationships, degree of participation in discussions, and relationship with father c. Predictor: (Constant); age, presence of children, presence of chronic diseases, and living with the elderly when young d. Predictor: (Constant); age, presence of children, presence of chronic illness, living with the elderly when young, self-esteem, continuity of self-concept, trust in people, sensitivity to criticism, depressive affect, dreaminess, psychosomatic symptoms, feeling threatened in interpersonal relationships, degree of participation in discussions, parental interest, relationship with father, and psychic isolation e. Predictor: (Constant); age, marital status, education level, presence of chronic diseases, presence of children, living with elderly when young, and region of residence f. Predictor: (Constant); age, marital status, educational level, presence of chronic disease, presence of children, living with the elderly when young, region of residence, trust in people, sensitivity to criticism, depressive affect, psychosomatic symptoms, feeling threatened in interpersonal relationships, parental interest, relationship with father, and psychic isolation Dependent Variable: *: Psychosocial loss; **: Physical change; ***: Psychosocial growth

Model	R	R^2^	Adjusted R^2^	Std. Error	Change Statistics				
					R^2^ change	F change	df1	df2	Sig. F change
1*	0.413^a^	0.171	0.151	5.96	0.171	8.811	6	257	0.000
2*	0.653^b^	0.427	0.389	5.05	0.256	11.022	10	247	0.000
1**	0.583^c^	0.340	0.330	4.71	0.340	33.314	4	259	0.000
2**	0.803^d^	0.645	0.622	3.54	0.305	17.692	12	247	0.000
1***	0.386^e^	0.149	0.123	3.74	0.149	5.591	8	255	0.000
2***	0.706^f^	0.498	0.465	2.92	0.349	21.437	8	247	0.000

When the analysis results for the psychosocial loss sub-dimension are analyzed, it is seen that the variables in the first model explain 17.1% of the psychosocial loss. In the second model, 42.7% of the variance is explained with the sub-dimensions of the RSES, which were included later. The variance explained in the second model is the variance explained by all independent variables (sociodemographic variables and sub-dimensions of the RSES). In order to see how much of the overall variance is explained by the identified sub-dimensions of the RSES, the R^2^ change value was examined on the second model, and it was seen that this value was 0.256. This value means that when sociodemographic variables are controlled, the variables of the identified sub-dimensions of the RSES explain 25.6% of the variance in psychosocial loss (R^2^ change=25.6; p<0.001).

When the analysis results for the physical change sub-dimension are analyzed, it is seen that the variables in the first model explain 34% of the physical change. In the second model, 64.5% of the variance is explained with the sub-dimensions of the RSES, which were included later. The variance explained in the second model is the variance explained by all independent variables (sociodemographic variables and RSES sub-dimensions). In order to see how much of the overall variance is explained by the identified sub-dimensions of the RSES, the R^2^ change value was examined on the second model, and it was seen that this value was 0.305. This value means that when sociodemographic variables were controlled, the variables of the identified sub-dimensions of the RSES explained 30.5% of the variance in physical change (R^2^ change=30.5; p<0.001).

When the analysis results for the psychosocial growth sub-dimension are analyzed, it is seen that the variables in the first model explain 14.9% of the psychosocial growth. In the second model, 49.8% of the variance was explained with the sub-dimensions of the RSES that were included later. The variance explained in the second model is the variance explained by all independent variables (sociodemographic variables and RSES sub-dimensions). In order to see how much of the overall variance is explained by the identified sub-dimensions of the RSES, the R^2^ change value was examined on the second model, and it was seen that this value was 0.349. This value means that when sociodemographic variables are controlled, the variables of the identified sub-dimensions of the RSES explain 34.9% of the variance in psychosocial growth (R^2^ change=34.9; p<0.001).

The results of the hierarchical multiple regression analysis of the psychosocial loss subscale of the AAQ are presented in Table 5. According to the results of the analysis presented in Table 5, two models significantly predicted psychosocial loss scores. When the independent variables in the first model are analyzed, it is seen that age (β=-0.162; p=0.018), marital status (β=0.158; p=0.007), and region of residence (β=0.168; p=0.009) have a predictive effect on psychosocial loss. In other words, being married and living in the city can be interpreted as positively affecting the attitude toward psychosocial loss, while increasing age affects it negatively. When the effects of age, marital status, education level, presence of chronic diseases, living with the elderly when young, and region of residence were controlled, it was observed that self-esteem (β=-0.234; p<0.001), depressive affect (β=-0.288; p=0.001), degree of participation in discussions (β=0.204; p=0.004), and relationship with father (β=-0.166; p=0.002) sub-dimensions in the second model had a predictive effect on psychosocial loss. In other words, it can be interpreted that low self-esteem, high depressive affect, and a more negative relationship with the father lead to negative shaping of attitudes in the field of psychosocial loss in the aging process.

**Table 5:** Results of a hierarchical multiple regression analysis for psychosocial loss Note: p<0.05: statistical significance level; dependent variable: psychosocial loss; t: independent samples t-test

Model		Unstandardized Coefficients		Standardized Coefficients	t-value	p-value
		B	Std. Error	Beta		
1	(Constant)	33.870	5.71	-	5.937	0.000
	Age	-0.167	0.07	-0.162	-2.383	0.018
	Marital status	2.718	1.01	0.158	2.706	0.007
	Education level	0.799	1.07	0.048	0.748	0.455
	Presence of chronic disease	-1.428	0.79	-0.110	-1.813	0.071
	Living with the old when you are young	1.805	1.05	0.113	1.714	0.088
	Region of residence	2.384	0.90	0.168	2.636	0.009
2	(Constant)	41.004	5.86	-	6.995	0.000
	Age	-0.097	0.07	-0.094	-1.359	0.175
	Marital status	3.095	0.91	0.180	3.406	0.001
	Education level	0.244	1.06	0.015	0.231	0.817
	Presence of chronic disease	-1.946	0.75	-0.150	-2.586	0.010
	Living with the old when you are young	-3.779	1.47	-0.236	-2.580	0.010
	Region of residence	2.368	0.94	0.167	2.529	0.012
	Self-Esteem	-2.433	0.63	-0.234	-3.891	0.000
	Continuity of self-concept	-0.583	0.29	-0.116	-1.955	0.052
	Trust in people	-0.420	0.64	-0.040	-0.652	0.515
	Sensitivity to criticism	-0.494	0.47	-0.070	-1.031	0.303
	Depressive affect	-1.464	0.45	-0.288	-3.282	0.001
	Dreaminess	-0.845	0.59	-0.098	-1.428	0.155
	Psychosomatic symptoms	0.105	0.18	0.044	0.582	0.561
	Feeling threatened in interpersonal relationships	-0.077	0.41	-0.014	-0.190	0.850
	Degree of participation in discussions	1.562	0.55	0.204	2.868	0.004
	Relationship with the father	-0.774	0.25	-0.166	-3.117	0.002

The results of the hierarchical multiple regression analysis of the physical change sub-dimension of the AAQ are given in Table 6. According to the results of the analysis presented in Table 6, two models significantly predicted physical change scores. When the independent variables in the first model are examined, it is seen that the presence of children (β=0.130; p=0.011), the presence of chronic illness (β=0.347; p<0.001), and the variable of living with the elderly when young (β=0.362; p<0.001) have a predictive effect on the attitude toward physical change. In other words, it can be interpreted that having children, having chronic diseases, and living with the elderly when young affect physical change positively. When the effect of age, presence of children, presence of chronic illness, and living with an elderly person when young is controlled, the continuity of self-concept (β=-0.114; p=0.015), sensitivity to criticism (β=-0.108; p=0.044), depressive affect (β=-0.429; p<0.001), which are included in the second model (β=-0.429; p<0.001), degree of participation in discussions (β=-0.156; p=0.003), relationship with the father (β=0.238; p<0.001), and psychic isolation (β=0.112; p=0.025) had a predictive effect on physical change. In other words, higher self-concept continuity, less sensitivity to criticism, less depressive affect, and a better ability to participate in discussions can be interpreted as positively affecting attitudes toward physical change in the aging process.

**Table 6:** Results of a hierarchical multiple regression analysis for physical change Note: p<0.05: statistical significance level; dependent variable: physical change; t: independent samples t-test

Model		Unstandardized Coefficients		Standardized Coefficients	t-value	p-value
		B	Std. Error	Beta		
1	(Constant)	14.672	4.25	-	3.453	0.001
	Age	-0.054	0.05	-0.059	-1.058	0.291
	Child presence	3.232	1.27	0.130	2.554	0.011
	Presence of chronic disease	4.013	0.61	0.347	6.663	0.000
	Living with the old when you are young	5.158	0.78	0.362	6.615	0.000
2	(Constant)	19.461	3.96	-	4.916	0.000
	Age	0.010	0.05	0.011	0.208	0.835
	Child presence	2.368	1.01	0.095	2.342	0.020
	Presence of chronic disease	3.156	0.52	0.273	6.019	0.000
	Living with the old when you are young	3.297	1.05	0.231	3.133	0.002
	Self-esteem	-0.783	0.44	-0.085	-1.787	0.075
	Continuity of self-concept	-0.511	0.21	-0.114	-2.457	0.015
	Trusting People	-0.064	0.46	-0.007	-0.139	0.889
	Sensitivity to criticism	-0.674	0.33	-0.108	-2.022	0.044
	Depressive affect	-1.944	0.29	-0.429	-6.639	0.000
	Dreaminess	0.022	0.41	0.003	0.053	0.958
	Psychosomatic symptoms	-0.132	0.12	-0.061	-1.132	0.259
	Feeling threatened in interpersonal relationships	-0.046	0.29	-0.009	-0.159	0.874
	Degree of participation in discussions	-1.067	0.36	-0.156	-2.986	0.003
	Parental interest	0.201	0.15	0.059	1.330	0.185
	Relationship with the father	0.989	0.17	0.238	5.698	0.000
	Psychic isolation	1.035	0.46	0.112	2.249	0.025

The results of the hierarchical multiple regression analysis of the psychosocial growth sub-dimension of the AAQ are given in Table 7. According to the results of the analysis presented in Table 7, both models significantly predicted psychosocial growth scores. When the independent variables in the first model are examined, it is seen that gender (β=-0.130; p=0.037), educational level (β=0.131; p=0.047), presence of chronic disease (β= -0.197; p=0.002), and living with the elderly when young (β=0.187; p=0.006) have a predictive effect on psychosocial growth. In other words, it can be interpreted that being a university graduate and living with the elderly when young affect the psychosocial growth attitude positively, while the female gender and the presence of chronic diseases affect the psychosocial growth attitude negatively. When the effects of age, gender, marital status, education level, presence of children, presence of chronic diseases, living with the elderly when young, and region of residence are controlled, the second model includes trust in people (β=-0.205; p=0.001), sensitivity to criticism (β=-0.313; p<0.001), parental care (β=0.562; p<0.001), relationship with the father (β=0.127; p=0.011), and psychic isolation (β=-0.222; p<0.001) in the second model had a predictive effect on psychosocial growth. In other words, a high sense of trust, low sensitivity to criticism, and low psychic isolation can be interpreted as positively affecting the attitude in the field of psychosocial growth in the aging process.

**Table 7:** Results of a hierarchical multiple regression analysis for psychosocial growth Note: p<0.05: statistical significance level; dependent variable: psychosocial growth; t: independent samples t-test

Model		Unstandardized Coefficients		Standardized Coefficients	t-value	p-value
		B	Std. Error	Beta		
1	(Constant)	21.927	3.72	-	5.893	0.000
	Age	0.024	0.05	0.037	0.522	0.602
	Gender	-1.080	0.52	-0.130	-2.095	0.037
	Marital status	0.803	0.76	0.076	1.054	0.293
	Education level	1.361	0.68	0.131	1.999	0.047
	Child presence	0.780	1.23	0.045	0.632	0.528
	Presence of chronic disease	-1.581	0.49	-0.197	-3.170	0.002
	Living with the old when you are young	1.854	0.67	0.187	2.750	0.006
	Region of residence	0.796	0.59	0.091	1.357	0.176
2	(Constant)	13.745	3.71	-	3.703	0.000
	Age	0.148	0.041	0.232	3.591	0.000
	Gender	0.879	0.49	0.103	1.764	0.079
	Marital status	0.038	0.64	0.004	0.059	0.953
	Education level	4.081	0.67	0.394	6.0.78	0.000
	Child presence	0.687	1.00	0.040	0.687	0.493
	Presence of chronic disease	-1.063	0.44	-0.133	-5.269	0.000
	Living with the old when you are young	2.930	0.80	0.296	3.645	0.000
	Region of residence	0.463	0.52	0.053	0.886	0.376
	Trust in people	-1.325	0.38	-0.205	-3.497	0.001
	Sensitivity to criticism	-1.358	0.25	-0.313	-5.269	0.000
	Depressive affect	-0.341	0.25	-0.108	-1.362	0.174
	Psychosomatic symptoms	0.000	0.11	0.000	0.003	0.998
	Feeling threatened in interpersonal relationships	0.129	0.23	0.037	0.560	0.576
	Parental Interest	1.325	0.14	0.562	9.444	0.000
	Relationship with the father	0.366	0.14	0.127	2.550	0.011
	Psychic isolation	-1.428	0.39	-0.222	-3.603	0.000

## Discussion

In our study, it was observed that self-esteem shaped from childhood to the present day was at least as effective as sociodemographic characteristics in the attitude toward aging.

It was found that attitudes toward psychosocial growth in the aging process were more positive in men, and attitudes toward psychosocial loss and physical change worsened with increasing age. Attitudes toward psychosocial loss were found to be more positive in those who were married and lived in the city; attitudes toward physical change were found to be more positive in those who had children, chronic illnesses, and experience of living with an elderly person when they were young; attitudes toward psychosocial growth were found to be more positive in university graduates and those who had experience of living with an elderly person when they were young, but more negative in those with a chronic illness.

In 2014, a thesis study was conducted with 200 patients over 65 years of age who applied to Başkent University Ankara Hospital outpatient clinics in four months and evaluated the attitude toward aging with the AAQ scale that we also used. Similar to our study, it was observed that the general attitude toward aging and attitude toward physical change were more positive in men [18]. This finding may be related to individuals' ability to have more access to social support systems or gender roles. In the same study, the study group was divided into 65-69 years of age, 70-74 years of age, and over 75 years of age when analyzing AAQ scores and age. It was found that the general attitude toward aging and attitude toward psychological loss were significantly more positive in the 65-69 age range than in the 70-74 age range [18]. Although this result was evaluated using a different method, it partially supports our results.

In a study conducted by Janečková et al. with 220 elderly people living in nursing homes, it was shown that the psychosocial loss sub-dimension of the perception of old age was negatively affected as the age progressed [19]. In our study, especially with the increase in the age of people, being withdrawn from the social environments caused by not being physically active as much as when they were young suggests that it negatively affects the attitude in this area. Again, in Sözen et al.'s study, similar to our study, it was found that the attitude toward psychosocial loss was higher in married people than in divorced and single people, although statistical significance could not be found. However, in contrast to our study, this attitude was significantly higher in widowed people than in married people [18].

In a thesis study conducted with 125 people over the age of 65 who applied to Istanbul University Cerrahpaşa Medical Faculty Hospital, Department of Internal Medicine, Division of Geriatrics Outpatient Clinic, it was shown that the attitude toward aging was more positive in married people, although it was not statistically significant, which was similar to the findings of our study [20]. In the same study, the mean scores of the elderly living in the city in the field of psychosocial loss were higher than the mean loss scores of the elderly living in towns and villages, supporting the findings of our study [20]. Today, while the number of people living in rural areas is decreasing, the number of people living in cities is increasing. Even if elderly people living in rural areas stay in contact with their acquaintances in the city, their ties with their social environment weaken over time. The fact that health services are more inadequate in these regions compared to large settlements leads to the fact that elderly people living alone in rural areas are deprived of support systems [21]. In our study, it is thought that the fact that individuals living in districts and villages had a more negative perception of old age in the sub-dimensions of psychosocial loss and physical change compared to those living in the city is due to these existing negativities. The fact that the attitudes toward psychosocial loss of those who are married and those who live in the city are more positive can be evaluated as marriage and city life strengthen social support mechanisms.

In Sözen et al.'s study, similar to our study, the total scores of AAQ, physical change, and psychological improvement scores were found to be significantly higher in university graduates. The study conducted by Rejeh et al. in 2016 found that physical change, psychosocial loss, and total AAQ score were significantly affected by educational status [22]. Individuals with a higher level of education have a more positive attitude toward the aging process and show a more positive approach in the psychosocial growth sub-dimension. Education contributes to the development of a more positive perspective on the aging process by enabling individuals to establish more active and healthy relationships with their social environment. Education, especially in the field of health, contributes significantly to the processes of socialization and information acquisition. It encourages people to gather information, evaluate it, and put it into practice. The impact of education on adults can be seen in all areas, and this impact is constantly growing in a positive direction. Education also provides positive effects on improving and protecting health in different ways [23]. For these reasons, in our study and previous studies, it is thought that a high level of education has a positive effect on the attitude toward aging.

When we look at the literature on the effect of the presence of a concomitant chronic disease on the attitude toward aging, Sözen et al. found that the attitudes toward physical change and the general attitude of the elderly with concomitant diseases were more negative [18]. This study may have concluded that attitudes were negatively affected due to the additional burden of comorbidities. However, in our study, while the presence of a chronic disease negatively affected the attitude toward psychosocial growth, it was found to have a positive effect on the attitude toward psychosocial loss. Having a chronic disease that starts before the person feels old, before experiencing the psychosocial loss brought by old age, and the fact that it continues gradually may be confronting the person with a habitual loss. This may lead to a more positive attitude toward the psychosocial loss that comes with aging.

On the other hand, it is thought that the burden brought by the additional disease may prevent the individual from giving positive answers to questions that evaluate psychosocial growth, such as "people can cope with life better as they get older, getting older is a privilege, there are many pleasant things about getting older, I want to pass on what I have gained with my experiences to young people, I am glad I lived, I believe that my life was useful." Chronic diseases may cause social isolation and loneliness by limiting individuals' participation in social activities. This may weaken the feeling that aging is a privilege or that experiences are valuable. Individuals with chronic illnesses may experience anxiety that their health will worsen in the future. This may lead to a feeling that sharing the experiences and knowledge of aging is not valuable and may negatively affect the individual's attitude toward psychosocial growth.

Our study found that attitudes toward psychosocial loss were more positive among older adults who had previously lived with older adults and had children. This finding suggests that social interactions and family ties play an important role in the aging process. Having lived with elderly individuals before may have enabled individuals to gain a broader perspective on the aging process and improve their coping skills with psychosocial losses. This may help individuals to be more prepared for the aging process and to face this process in a more positive way.

Similarly, having children has a positive effect on individuals' attitudes toward psychosocial losses. Individuals with children can strengthen their social support mechanisms by assuming more active roles within the family. This may increase their ability to cope with psychosocial losses in the aging process. Strong family interactions and support systems contribute to older individuals feeling more secure and supported. Similar findings are also found in the literature [21,24]. It has been reported that individuals with strong social support systems have a more positive approach to the aging process and cope better with psychosocial losses. Our findings emphasize the importance of social and familial ties in the aging process.

It was found that high self-esteem, continuity of self-concept, and low depressive mood and psychosomatic symptoms, as determined by the sub-dimensions of the RSES, positively affected the attitude of psychosocial loss in the aging process. Although each sub-dimension was evaluated separately, the findings generally showed that people with high self-esteem had a more positive attitude toward aging. When the factors predicting the attitude toward aging were examined, it was found that 25.6% of the attitude toward psychosocial loss was explained by self-esteem, self-concept continuity, trust in people, sensitivity to criticism, depressive affect, dreaminess, psychosomatic symptoms, feeling threatened in interpersonal relationships, degree of participation in discussions, and relationship with father, which were determined by the sub-dimensions of RSES; 30.5% of the attitude toward physical change was explained by self-esteem, self-concept continuity, trust in people, sensitivity to criticism, depressive affect, imagination, psychosomatic symptoms, feeling threatened in interpersonal relationships, degree of participation in discussions, parental interest, relationship with the father, and psychic isolation determined by the sub-dimensions of RSES; 34.9% of the attitude toward psychosocial growth was explained by trust in people, sensitivity to criticism, depressive affect, psychosomatic symptoms, feeling threatened in interpersonal relationships, parental interest, relationship with father, and psychic isolation, which were determined by the sub-dimensions of RSES.

Erikson (1982) stated that self-integrity for older individuals is equally important as the basic sense of trust in infancy [25]. According to him, life is a process in which each stage is a preparation for the next stage. In this context, the human life cycle is considered a gradual process of continuous development and change. In this process, infancy, childhood, adolescence, and adulthood are considered to be stages of biopsychosocial growth, while old age is generally seen as a stage of decline. These declines in old age can be slowed or delayed by scientific and technological developments, but the existence of physiological declines caused by diseases and physiological changes cannot be denied. However, despite the physiologic decline, old age can be considered not only a period of decline but also a period in which psychosocial growth continues [25]. Therefore, in our study, we investigated the effect of experiences in interpersonal relationships acquired from childhood to the present day, the sense of trust, the discussion environments they experienced and participated in, the depressive states they experienced, psychosomatization, and the self-esteem formed by the interest of their families on the attitude toward aging.

Studies show that people with high self-esteem are healthy, productive, happy, and success-oriented individuals; they make longer efforts to cope with difficulties, sleep better at night, and are more insensitive to the pressures of their peers [26]. Coleman explained that individuals with low self-esteem are pessimistic, have negative feelings about the future, and are failure-oriented [27]. As a result of the research, Kassin stated that individuals with low self-esteem have expectations of failure, may ignore important points, fail to achieve success, make less effort, tend to be irritable, and blame themselves for being incompetent and worthless [28].

In a study conducted in Korea with 211 elderly people, where attitudes toward aging were assessed using the AAQ used in our study, it was shown that the elderly's attitudes toward successful aging were directly and indirectly affected by psychosocial factors such as sense of community, depression, and interpersonal needs and that the need for interpersonal relationships had a significant mediating effect on the relationships between attitudes toward aging, sense of community, and depression [29]. This means that depression and insufficient fulfillment of interpersonal needs in the elderly have a negative effect on the attitude toward aging.

The relationships that an individual establishes throughout life are important determinants of aging. In a study conducted by Moser et al. in 2011, it was found that single people had an effect on negative perceptions of old age [30]. A study conducted by Shenkin et al. showed that living alone was a significant independent determinant of psychosocial growth [31]. In our study, especially the psychosocial growth sub-dimension was found to be associated with psychic isolation. In a stratified, randomized community longitudinal study of 300 adults between 49 and 51 years of age in New Zealand, attitudes toward aging were measured with the AAQ used in our study. Comorbid chronic diseases, depression, and sociodemographic data were also assessed. It has been previously shown that individuals may have positive attitudes toward aging when they have poorer physical health, but that attitudes are more negatively affected in the presence of low mood, and that current depression has the strongest association with attitudes toward aging and mediates the relationship between attitudes toward healthy aging [32].

In the United States, a 10-year longitudinal study examined both the subjective age and the chronological age of people in relation to their well-being. Positive attitudes toward aging were associated with better psychological well-being, even when the subjective age was older than the chronological age. A positive attitude toward their own aging among older adults has been interpreted as serving as a psychological resource to mitigate the effects of the aging process [33]. Many studies also show that a positive perception of aging improves quality of life [34]. Despite the evidence demonstrating the importance of attitudes toward aging, there is a limited number of studies investigating the factors that may contribute to older adults' attitudes toward aging. Therefore, we think that it is important to determine the place of self-esteem, which is formed by experiences from childhood to old age, in the attitude toward aging.

In most of the studies on life attitude in the literature, the elderly were not examined psychiatrically before inclusion in the study, and psychiatric disorders were not excluded. The inclusion of our participants after a detailed psychiatric evaluation is a strength of our study. Studies evaluating self-esteem have mostly used the RSES short form consisting of 10 questions. In our study, we used the long form of the scale consisting of 63 questions and 12 subscales to evaluate the participants in more detail. As far as we have examined, this is the first study to examine the relationship between self-esteem and attitude toward old age, which makes our study unique.

However, our study also has some limitations. Since the number of childless elderly people in the sample was relatively small, the assessment of the effect of the presence of children may not have been optimal. The fact that most of the sample is female may create a bias for our results. It covers older adults living in a medium-sized city in Turkey and is not a representative sample, so the generalizability of the results of this study is limited. Future large-scale and multicenter studies are needed. With multicenter studies, demographic diversity can be increased with participants from different geographical regions. This way, the data obtained can represent a larger population and have higher generalizability [35]. In addition, caution should be exercised in interpreting the results, as various characteristics of older adults that may act as exogenous variables were not controlled for. No research has addressed and evaluated the relationship between attitude toward aging and self-esteem in older adults. Therefore, it was not possible to compare the results of this study with other studies. Within the framework of this limitation, the results of the study were compared with the findings of similar studies.

Considering the results of our study, it is important to encourage positive experiences and achievements that will increase the self-esteem of individuals so that the aging process can be experienced as a positive experience. At every stage of life, supportive programs and psychological counseling services should be provided to strengthen the self-esteem of individuals with low self-esteem. In addition, maintaining healthy family relationships is possible by strengthening communication within the family and keeping relationships with parents intact. Promoting positive emotions such as trust, love, and support among family members ensures that individuals have strong social support networks in old age. In addition, educational programs should be organized to develop positive attitudes about aging and old age throughout society, and a conscious and aware approach should be adopted regarding the aging process. In this way, we think that it will be possible for individuals to lead a healthier and higher quality life in old age, both psychologically and socially.

## Conclusions

Our study found that people with high self-esteem had a more positive attitude toward aging. When the effect of sociodemographic data was controlled, it was seen that approximately one-fourth of the attitudes toward psychosocial loss during the aging process could be explained by self-esteem. It was also observed that approximately one-third of the attitude toward physical change could be explained by self-esteem. Lastly, it was found that approximately one-third of the attitude toward psychosocial growth could be explained by self-esteem. The results of our study show that self-esteem and self-confidence developed at this age can help the individual to approach life with a more positive perspective in the following years. Considering that the basis of healthy aging is perhaps determined by personality traits and self-esteem established in childhood and adolescence, we believe that our study's findings are valuable in forming the basis of efforts to increase the well-being of the growing elderly population.
